# Risk factors for severe systemic sting reactions in wasp (*Vespula* spp.) and honeybee (*Apis mellifera*) venom allergic patients

**DOI:** 10.1186/s13601-019-0292-5

**Published:** 2019-10-11

**Authors:** Danielle Fehr, Sara Micaletto, Thomas Moehr, Peter Schmid-Grendelmeier

**Affiliations:** 1 Allergy Unit, Department of Dermatology, University Hospital Zurich, Gloriastrasse 31, 8091 Zurich, Switzerland; 2 B,S,S. Economic Consultants, Aeschengraben 9, 4051 Basel, Switzerland

**Keywords:** Allergy, Bees, Hymenoptera venom, Risk factors, Wasps

## Abstract

**Background:**

Hymenoptera stings are a major cause of anaphylaxis. Various risk factors are discussed in literature. This study aims to investigate potential risk factors for severe sting reactions in wasp (*Vespula* spp.) and honeybee (*Apis mellifera*) venom allergic patients and analyses the correlation between diagnostic test results and the severity of the allergic reaction.

**Methods:**

480 patients suffering from wasp or honeybee venom allergy were included in this retrospective case series. Only individuals allergic to *Vespula* spp. but not to other vespids such as* Polistes* were considered. The severity of their systemic field sting reaction was analysed with regard to the amount of specific IgE antibodies to whole venom extracts and to major allergens of honeybee and/or wasp venom. Furthermore, the following potential risk factors for severe sting reactions were examined: age, sex, latency time, skin symptoms, baseline serum tryptase levels and the concentration of venom inducing a positive intracutaneous test.

**Results:**

The two following indicators for severe systemic sting reactions in honeybee and wasp venom allergic patients have been identified: a short latency time and the absence of skin symptoms. The patient’s age and baseline serum tryptase levels have been found to positively correlate with the grade of the sting reaction only in individuals allergic to wasp venom. No correlation could be found between the degree of sensitisation and the severity of the allergic reaction. Neither the amount of specific IgE antibodies to whole venom extracts nor to major allergens were significantly associated with the severity of the sting reaction.

**Conclusion:**

The clinical history is essential for the allergological workup and therapeutic decision on Hymenoptera venom allergies. A short latency time and the absence of skin symptoms are indicators for severe systemic sting reactions, followed by the patient’s age and baseline serum tryptase levels.

## Background

Hymenoptera stings are among the most common reasons for anaphylaxis [1, 2]. In Europe, between 0.3 and 7.5% of the general population are affected by systemic anaphylactic sting reactions [3]. Different clinical manifestations are possible, ranging from a mere skin reaction (flush, pruritus, urticaria, angioedema) to gastro-intestinal or respiratory symptoms, cardiovascular involvement or in some cases even death [4].

Different studies have investigated risk factors for severe sting reactions. Among others, the following aspects are mentioned in literature: male sex, older age, a large number of stings, a short time interval between stings, the localisation of the sting, the absence of skin symptoms, high baseline serum tryptase (BST) levels as well as cardiovascular comorbidity and medication [3, 5–11]. However, little research has been conducted to evaluate potential differences between wasp (*Vespula* spp.) and honeybee (*Apis mellifera*) venom allergic patients.

The diagnostic workup of patients with suspected Hymenoptera venom allergy includes a comprehensive clinical history, skin tests and the measurement of specific IgE antibodies (sIgE) to whole venom preparations. Additional in vitro tests can be necessary in case of unexpected negative results. BST levels are evaluated in case of systemic anaphylaxis [12, 13]. In recent years, diagnostic accuracy of insect sting allergies has been improved by using molecular diagnostics. SIgE to major allergens such as phospholipase A2 (rApi m 1) in honeybee venom or phospholipase A1 (rVes v 1) and antigen 5 (rVes v 5) in wasp venom can help to distinguish genuine double sensitisation from cross-reactivity [13–15].

We undertook a consecutive retrospective case series to investigate the correlation between the amount of sIgE to whole venom extracts respectively to major allergens and the severity of the systemic sting reaction (SSR). An additional aim of this study was to evaluate the following potential risk factors for severe SSR in both wasp and honeybee venom allergic patients: age, sex, latency time, skin symptoms, BST levels and the concentration of venom inducing a positive intracutaneous test.

## Methods

### Patients

This consecutive retrospective case series included 480 patients suffering from *Vespula* spp. (hereafter referred to as wasp) or *Apis mellifera* (hereafter referred to as honeybee) venom allergy. Patients allergic to other vespids such as* Polistes*,* Vespa crabro* or *Dolichovespula* were not included. From January 2010 to December 2016, the respective individuals were referred to the Department of Dermatology of the University Hospital Zurich in view of a venom immunotherapy (VIT). As a consequence, all patients met the criteria of the European Academy of Allergy and Immunology for the initiation of VIT. VIT is indicated in sensitised individuals who suffered a SSR exceeding generalised cutaneous symptoms. It is also recommended if it improves quality of life in adult patients with only generalised skin reactions [16]. Individuals were included only if the SSR occurred following a Hymenoptera field sting. Patients sensitised to both wasp and honeybee venom were excluded. In addition, only laboratory values measured prior to the initiation of VIT were taken into account.

In patients with elevated BST levels (n = 35) a detailed clinical history and careful inspection of the skin was performed to detect any signs of underlying mastocytosis. If BST was > 20 μg/l (n = 14), an osteodensitometry was initiated to detect an associated osteoporosis; if symptoms highly suspicious for systemic mastocytosis were reported or BST was > 30 μg/l, a bone marrow aspiration was performed (n = 10). Detection of c-KIT mutation (D816V) in peripheral blood would have been another useful diagnostic test in this patients but was not available at the time of the data collection.

### Classification of sting reactions

The systemic reactions to Hymenoptera stings were classified according to H. L. Mueller [17] on a scale from I to IV (Table 1). Details about the allergic episode were obtained from medical reports and letters of referral.

**Table 1:** Classification of systemic sting reactions (after H. L. Mueller [16], modified by U. R. Mueller [4])

Grade	Symptoms
I	Generalised urticaria, itching, malaise, anxiety
II	Any of the above plus two or more of the following: angioedema (grade II also if alone), constriction in chest, nausea, vomiting, diarrhoea, abdominal pain, dizziness
III	Any of the above plus two or more of the following: dyspnoea, wheezing, stridor (any of these alone are grade III), dysphagia, dysarthria, hoarseness, weakness, confusion, feeling of impending disaster
IV	Any of the above plus two or more of the following: fall in blood pressure, collapse, loss of consciousness, incontinence (urine, stool), cyanosis

### Intracutaneous skin tests

Intracutaneous tests were performed with 0.02 ml doses of wasp and honeybee venom extracts (Pharmalgen ALK-Abelló aqueous extracts, Hørsholm, Denmark). Beginning with a concentration of 0.00001 µg/ml, the injected concentration was increased by a factor of 10 every 15 min. If a wheal with a diameter of 3 mm or more occurred within those 15 min, the test was considered positive and no further application was made. If no reaction occurred until the concentration of 1 µg/ml, the test was stopped and considered negative. NaCl 0.9% served as negative and histamine as positive control. Skin test results obtained by a referring physician were not taken into account for statistical analyses.

### Laboratory

BST as well as sIgE levels (honeybee i1, rApi m 1, wasp i3, rVes v 1, rVes v 5) were measured with the ImmunoCAP-System™ (Thermo Fisher Scientific, Waltham, USA). The tests were performed according to the manufacturer’s instructions. Only laboratory values determined before the beginning of VIT were included in the dataset. Values established by referring hospitals were not considered in statistical analyses due to potential variations in measurements.

### Statistics

STATA Version 13.0 was used for statistical analyses. A p-value of less than 0.05 was considered as being statistically significant. The main outcome variable was the grade of SSR. Since it is ordinally defined on a scale from one to four, ordinal logistic regressions were used. The grade was separately regressed on each potential risk factor, always controlling for the patient’s age and sex. The underlying proportional odds assumption was tested by Brant’s Wald test [18]. The assumption was rejected two times: in the regression on age for honeybee venom allergic patients and on BST for wasp venom allergic patients. However, the insignificance of the former and the significance of the latter were confirmed by the Jonckheere-Terpstra test. Regarding BST levels, a multivariate logistic regression on a grade IV dummy variable could additionally confirm the positive correlation.

For analyses with a nominally scaled outcome variable (BST level or concentration of sIgE) Spearman’s non-parametric rank correlation tests were conducted. Regarding the presence of an elevated BST level, patients were compared by a Chi square contingency table.

## Results

### Patient characteristics

**Fig. 1:**
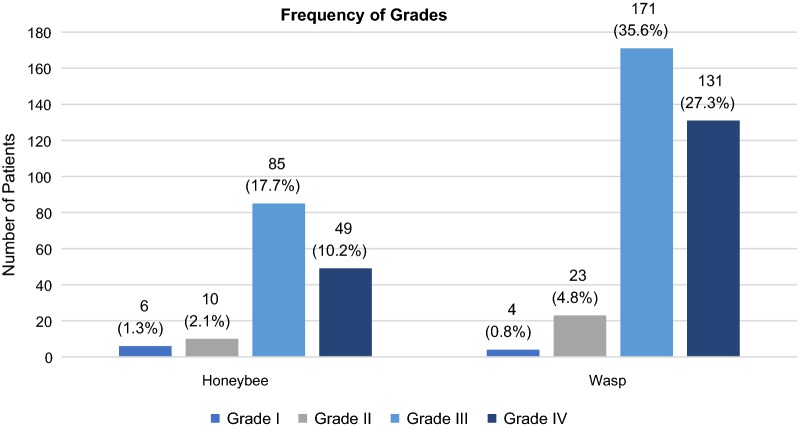
Frequency of grades according to each insect

A total of 480 patients were included in this study. Consisting of 243 males (50.6%) and 237 females (49.4%), the data were almost equally balanced in terms of sex. Patients were between 6 and 76 years old, the average age being 41.6 years. 180 patients (37.5%) suffered a grade IV sting reaction, 256 reactions (53.3%) were classified as grade III, 33 reactions (6.9%) as grade II and only 10 patients (2.1%) had a grade I sting reaction. In one case, the information about the allergic reaction was contradictory and therefore, the grade could not be determined. The frequency of the grades according to each insect is shown in Fig. 1. In 330 patients (68.8%), the culprit insect was a wasp (*Vespula* spp.) and in 150 patients (31.3%) a honeybee (*Apis mellifera*). On average, it took 13 min to the onset of symptoms. 90% of the reactions occurred within 30 min after the sting. Cutaneous manifestations were present in 326 patients (67.9%), 99 individuals (20.6%) did not show any skin symptoms and for the remaining ones, the information was missing. BST levels were measured in 437 patients of whom 35 (8.0%) had an elevated BST level (defined as ≥ 11.4 µg/l). An overview of the patient characteristics is given in Table 2.

**Table 2:** Patient characteristics (percentage or standard deviation in parentheses)

Number of patients	Total	Wasp venom allergy	Honeybee venom allergy
	480	330	150
Sex			
Female	237 (49.4%)	163 (49.4%)	74 (49.3%)
Male	243 (50.6%)	167 (50.6%)	76 (50.7%)
Age (years)			
Mean	41.6	42.5	39.3
Range	6–76	6–76	9–69
Severity of the allergic reaction			
Grade I	10 (2.1%)	4 (1.2%)	6 (4.0%)
Grade II	33 (6.9%)	23 (7.0%)	10 (6.7%)
Grade III	256 (53.3%)	171 (51.8%)	85 (56.7%)
Grade IV	180 (37.5%)	131 (39.7%)	49 (32.7%)
Latency time (min)			
Mean	13.0 (± 14.9)	12.4 (± 13.5)	14.7 (± 17.9)
Cutaneous symptoms			
Yes	326 (67.9%)	224 (67.9%)	102 (68.0%)
No	99 (20.6%)	70 (21.2%)	29 (19.3%)
Missing	55 (11.5%)	36 (10.9%)	19 (12.7%)
Mean sIgE (kU/l)			
To honeybee venom (i1)	6.07 (± 15.5)	1.45 (± 5.5)	17.31 (± 23.9)
To rApi m 1	2.30 (± 10.2)	0.11 (± 0.3)	7.86 (± 18.1)
To wasp venom (i3)	9.13 (± 21.4)	12.28 (± 24.6)	1.34 (± 2.8)
To rVes v 1	5.71 (± 21.0)	7.59 (± 24.2)	0.42 (± 1.3)
To rVes v 5	7.34 (± 18.1)	9.80 (± 20.7)	0.77 (± 2.8)
BST level			
Measured	437	307	130
Normal (< 11.4 µg/l)	402 (92.0%)	277 (90.2%)	125 (96.2%)
Elevated (≥ 11.4 µg/l)	35 (8.0%)	30 (9.8%)	5 (3.8%)
Intracutaneous test			
Performed	428	300	128
0.00001 µg/ml	66 (15.4%)	40 (13.3%)	26 (20.3%)
0.0001 µg/ml	3 (0.7%)	3 (1.0%)	0 (0.0%)
0.001 µg/ml	69 (16.1%)	52 (17.3%)	17 (13.3%)
0.01 µg/ml	150 (35.0%)	111 (37.0%)	39 (30.5%)
0.1 µg/ml	98 (22.9%)	70 (23.3%)	28 (21.9%)
1 µg/ml	35 (8.2%)	22 (7.3%)	13 (10.2%)
Negative	7 (1.6%)	2 (0.7%)	5 (3.9%)

### Risk factors for a severe sting reaction

Whether the patient was stung by a honeybee or a wasp did not directly influence the severity of SSR (controlling for age and sex, p = 0.36). However, the culprit insect had an indirect effect by changing the significance of potential risk factors. Therefore, the same statistical analyses have been carried out for both Hymenoptera separately. The p-values of the ordinal logistic regressions are summarised in Table 3.

**Table 3:** P-values of the ordinal logistic regressions controlling for age and sex

Variables	Wasp Grade	Honeybee Grade
Age	< 0.01*	0.48
Male	0.16	0.34
Latency time	0.04*	0.02*
Cutaneous symptoms	< 0.01*	0.02*
i3	0.25	0.63
rVes v 1	0.72	0.41
rVes v 5	0.26	0.09
i1	0.32	0.76
rApi m 1	0.96	0.46
BST level	0.045*	0.15
Intracutaneous test	0.73	0.58

* p < 0.05

#### Wasp venom allergic patients

**Fig. 2:**
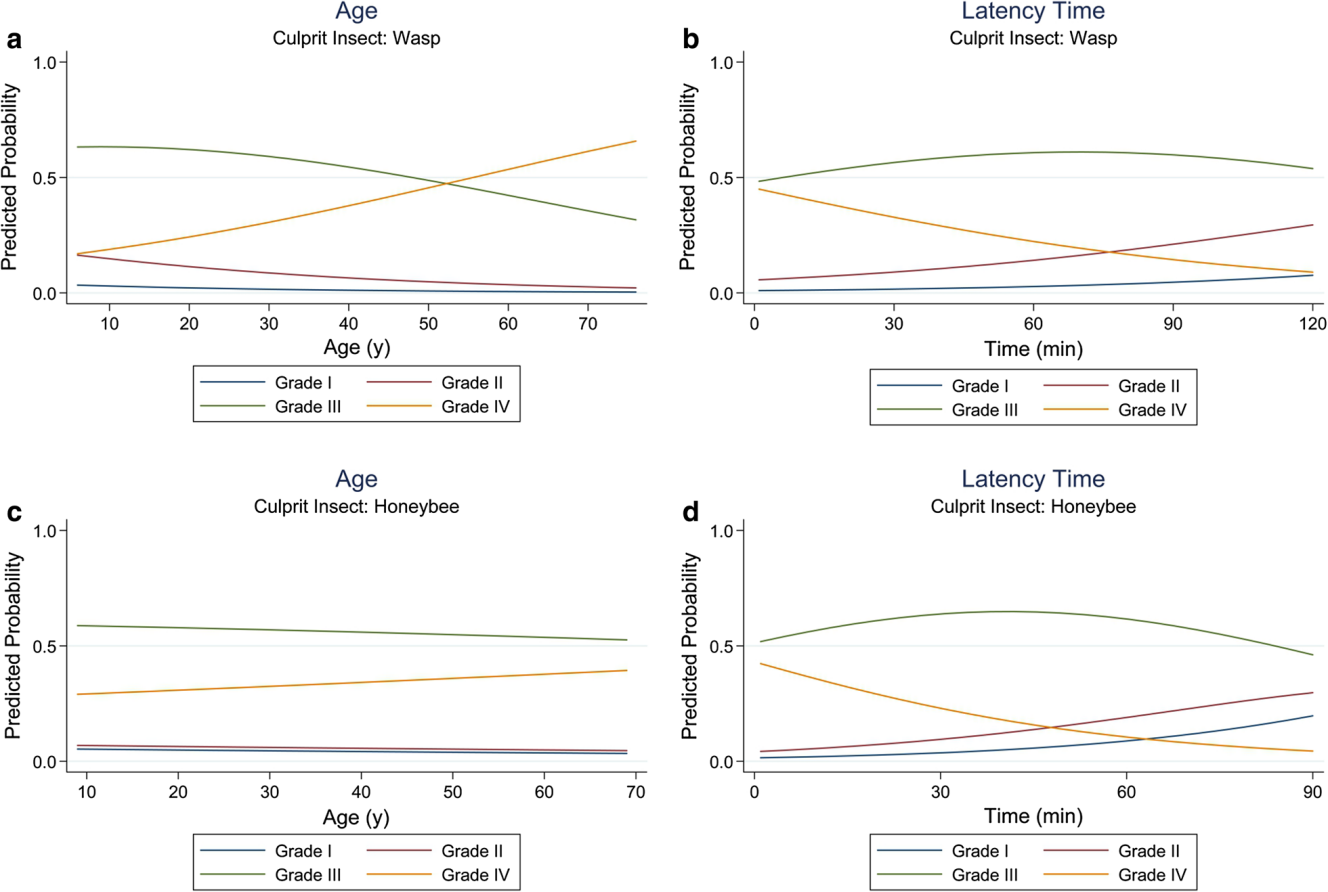
**a** Association between age and grade for wasp venom allergic patients. **b** Association between latency time and grade for wasp venom allergic patients. **c** Association between age and grade for honeybee venom allergic patients. **d** Association between latency time and grade for honeybee venom allergic patients

Only patients suffering from an allergy to* Vespula* spp. were included in this study, while individuals allergic to other vespids such as* Polistes*,* Vespa crabro* or* Dolichovespula* were not taken into account. The patient’s age positively correlated with the severity of SSR (p < 0.01). Thus, the older the patient is, the more severe the allergic reaction tends to be (Fig. 2a). Furthermore, a statistically significant positive correlation between the individual’s age and the measured BST level has been identified (p < 0.01). In contrast, the patient’s sex did not have any influence on the degree of clinical reactivity (p = 0.16).

Another indicator for severe SSR was a short latency time (Fig. 2b). On average, the faster the allergic reaction occurred after the sting, the more severe it was (p = 0.04).

Along with older age and a short latency time, the absence of skin symptoms has been found to be a risk factor for severe sting reactions. There was a significant positive correlation between the nonappearance of cutaneous symptoms (such as flush, pruritus, urticaria or angioedema) and the severity of reaction (p < 0.01). In addition, the absence of skin manifestations was associated with high BST levels (p = 0.01).

BST levels were determined in 307 patients suffering from an allergy to wasp venom. Analyses showed a statistically significant positive correlation between the degree of severity and the BST level (p = 0.045).

There was no significant association between the amount of sIgE to wasp venom (i3) and the severity of SSR (p = 0.25). Likewise, no correlation between sIgE to major allergens (rVes v 1, rVes v 5) and the grade could be found (p = 0.72 and p = 0.26).

Intracutaneous tests were undertaken on 300 patients allergic to wasp venom. Analyses could not identify a statistically significant relationship between the venom concentration required for a positive test result and the severity of SSR (p = 0.73). Yet, a strong correlation between the intracutaneous test result and the amount of sIgE to wasp venom was detected (p < 0.01).

#### Honeybee venom allergic patients

When it comes to the patient’s age, there is a difference between patients suffering from an allergy to honeybee venom compared to those allergic to wasp venom. Concerning honeybees, there was no statistically significant correlation between the individual’s age and the grade of SSR (p = 0.48, Fig. 2c). The patient’s sex was again not associated with the severity of SSR (p = 0.34).

It was possible to identify the two following risk factors for severe SSR in individuals allergic to honeybee venom: a short latency time and the absence of skin symptoms. Generally, the sting reaction was more severe if the time until the onset of symptoms was shorter (p = 0.02, Fig. 2d). Skin symptoms were inversely related to the degree of severity as well (p = 0.02). Hence, if no cutaneous manifestations occurred, the average grade was higher. In contrast to wasp venom allergic patients, there was no statistically significant correlation between the presence of skin symptoms and the measured BST level (p = 0.46).

Five honeybee venom allergic individuals out of 130 had an elevated BST level (3.8%). In contrast to the patients allergic to wasp venom, the concentration of BST did not significantly correlate with the grade (p = 0.15). Notwithstanding, the BST level has been found to increase with age (p < 0.01).

The concentration of sIgE to the whole venom preparation (i1) and to the major allergen (rApi m 1) did not significantly correlate with the grade of SSR (p = 0.76 and p = 0.46).

As was the case for wasp venom allergic patients, the result of the intracutaneous test was not significantly associated with the severity of SSR (p = 0.58). Nevertheless, statistical analyses showed a strong relation between the test result and the amount of sIgE to honeybee venom (p < 0.01).

## Discussion

The present study was designed to investigate the significance of different laboratory values and potential risk factors to predict the severity of a SSR following a Hymenoptera field sting. Our findings support the assertion that the degree of sensitisation does not correlate with the severity of the sting reaction [3, 19–27]. Neither the concentration of venom inducing a positive intracutaneous test, nor the amount of sIgE to whole venom preparations or to venom components were significantly associated with the grade of SSR. Given the fact that asymptomatic sensitisation is present in up to 40.7% of the population [19, 28], this is not unexpected. However, there was a statistically significant positive correlation between the skin test reactivity and the level of venom-specific IgE.

We found the following risk factors for severe SSR in wasp venom allergic patients: a short latency time, the absence of skin symptoms, older age and high BST levels. Interestingly, our results varied depending on the culprit insect. For patients allergic to honeybee venom, only the absence of cutaneous symptoms and a short latency time could be established as risk factors.

### Latency time

Usually, a shorter time interval between the sting and the beginning of symptoms indicates a more severe SSR. Similar to this finding, Stoevesandt et al. [9] retrospectively identified a latency time of less than 5 min as an indicator for severe SSR. In a prospective study evaluating risk factors by means of a sting challenge, the latency time also correlated negatively with the severity of reaction [20]. In our study, 75% of the grade IV reactions occurred during the first 10 min after the sting, the median time interval being 5 min. This is consistent with Sturm et al. [29] who observed that cardiovascular symptoms usually appear within 10 min.

### Cutaneous symptoms

The absence of skin symptoms (flush, pruritus, urticaria, angioedema) is significantly associated with severe SSR. This statement could be established for both wasp and honeybee venom allergic patients and is in line with previous findings [9]. In literature, the nonappearance of cutaneous manifestations has been described as a typical observation in patients suffering from severe cardiovascular symptoms after a Hymenoptera sting [29]. Concerning patients allergic to wasp venom, we could even observe a statistically significant association between the lack of skin involvement and high BST levels. Previous studies suggested that measurement of BST levels and medical clarification of possible clonal mast cell disorders are of great importance in patients with severe SSR who did not report any skin symptoms [9, 14, 30, 31]. We support this recommendation especially in regard to wasp venom allergic patients.

There are two possible explanations for the absence of skin symptoms in severe SSR. First, there could be a recall bias. Patients focus on the most threatening symptom such as loss of consciousness and do not remember the less severe symptoms. However, witnesses of the SSR or medical staff often confirm the lack of cutaneous involvement [9, 25]. Second, epinephrine is set free to counteract in patients suffering from circulatory symptoms. This hormone induces a peripheral vasoconstriction and thus conceivably inhibits the development of skin symptoms [9, 29, 30].

### Age

Concerning patients allergic to wasp venom, there was a statistically significant correlation between the age and the severity of SSR. Several studies have already described this age-related increase in severity [5, 8, 9, 20, 24, 32–34]. A potential reason for the greater risk of severe SSR (or even death [3, 10]) with increasing age is an impaired cardiovascular and/or pulmonary function due to pre-existing diseases [5, 9, 35]. Not only does this lead to a reduced potential to compensate and recover but also to the intake of medication such as ACE inhibitors or beta-blockers, which in turn are discussed as risk factors for severe allergic reactions [34, 36]. As recommended by the European Academy of Allergy and Immunology, physicians should consider a long-term or lifelong VIT in older patients [37]. Surprisingly, no statistically significant association between the patient’s age and the grade of SSR could be found for honeybee venom allergic individuals. There is a need for further research to elaborate on the reasons behind this finding.

### BST levels

Our analyses revealed that in patients allergic to wasp venom, higher BST levels were significantly associated with more severe SSR. The relation between BST levels and degree of severity has already been investigated in multiple other studies. Most of them did find a statistically significant positive association [5, 8, 9, 24, 34], whereas Sturm et al. [29] did not observe an influence of BST levels on the severity of the sting reaction. The finding that BST levels rise with increasing age is more controversial and needs further clarification. While Haeberli et al. [24] and Stoevesandt et al. [9] did not detect any association, Kucharewicz et al. [32], Blum et al. [5] and Guenova et al. [8] found a statistically significant one.

The fact that the correlation between BST levels and the severity of SSR did not reach statistical significance in honeybee venom allergic patients potentially stems from the smaller number of observations (n = 130 for honeybees, n = 307 for wasps). It is, however, interesting that among the individuals allergic to honeybee venom, only five out of 130 (3.8%) had an elevated BST level, whereas the number was significantly higher in wasp venom allergic patients (30 out of 307, 9.8%). Hence, 85.7% of the patients with increased BST levels were allergic to wasp venom. In the study of Haeberli et al., this percentage was similar (75%). They hypothesised that wasp venom contained peptides which are stronger activators of mast cells than those in honeybee venom. Thus, wasp venom could intensify the IgE mediated response more easily. As a consequence, mastocytosis would be more relevant in aggravating the sting reaction in wasp than in honeybee venom allergic patients [24]. However, further research is needed to examine this theory.

## Limitations

The first limitation of our research results from the retrospective design of the study. This implicates that we had to rely on the patient’s memory and accept a potential recall bias. However, we preferred the evaluation of past field stings because of the danger of provoking serious reactions inherent in sting challenges. The second limitation is that the time interval between the sting and the diagnostic workup varied, as the patients did not always search medical aid immediately.

## Conclusions

The degree of sensitisation does not indicate the severity of a previous sting reaction. Therefore, a comprehensive clinical history with focus on indicators for severe sting reactions is indispensable. The absence of skin symptoms and a short latency time are associated with severe SSR in both wasp and honeybee venom allergic patients. The patient’s age and BST levels significantly correlate with the grade in patients allergic to wasp venom only.

## Data Availability

The datasets generated and analysed during the current study are available from the corresponding author on reasonable request.

## Abbreviations

BST: baseline serum tryptase
SSR: systemic sting reaction
sIgE: specific IgE antibodies
VIT: venom immunotherapy
